# Spatial Distribution, Chemical Fraction and Fuzzy Comprehensive Risk Assessment of Heavy Metals in Surface Sediments from the Honghu Lake, China

**DOI:** 10.3390/ijerph15020207

**Published:** 2018-01-26

**Authors:** Fei Li, Minsi Xiao, Jingdong Zhang, Chaoyang Liu, Zhenzhen Qiu, Ying Cai

**Affiliations:** 1 Research Center for Environment and Health, Zhongnan University of Economics and Law, Wuhan 430073, China; lifei@zuel.edu.cn (F.L.); msxiao@zuel.edu.cn (M.X.); lcy@zuel.edu.cn (C.L.); zzqiu@zuel.edu.cn (Z.Q.); 1993cy@zuel.edu.cn (Y.C.); 2 School of Information and Safety Engineering, Zhongnan University of Economics and Law, Wuhan 430073, China

**Keywords:** surface sediments, heavy metals, spatial distribution, chemical fraction, fuzzy comprehensive risk assessment, Honghu Lake

## Abstract

Spatial concentrations and chemical fractions of heavy metals (Cr, Cu, Pb, Zn and Cd) in 16 sampling sites from the Honghu Lake were investigated using an atomic absorption spectrophotometer and optimized BCR (the European Community Bureau of Reference) three-stage extraction procedure. Compared with the corresponding probable effect levels (PELs), adverse biological effects of the studied five sediment metals decreased in the sequence of Cr > Cu > Zn > Pb > Cd. Geo-accumulation index (*I_geo_*) values for Cr, Cu, Pb and Zn in each sampling site were at un-contamination level, while the values for Cd varied from un-contamination level to moderate contamination level. Spatially, the enrichment degree of Cd in lower part of the South Lake, the west part of the North Lake and the outlet were higher than the other parts of Honghu Lake. For metal chemical fractions, the proportions of the acid-extractable fraction of five metal contents were in the descending order: Cd, Cu, Zn, Pb and Cr. Cd had the highest bioaccessibility. Being the above indexes focused always on heavy metals’ total content or chemical fraction in deterministic assessment system, which may confuse decision makers, the fuzzy comprehensive risk assessment method was established based on PEI (Potential ecological risk index), RAC (Risk assessment code) and fuzzy theory. Average comprehensive risks of heavy metals in sediments revealed the following orders: Cd (considerable risk) > Cu (moderate risk) > Zn (low risk) > Pb > Cr. Thus, Cd and Cu were determined as the pollutants of most concern. The central part of South Honghu Lake (S4, S5, S6, S9, S12 and S14), east part of the North Honghu Lake (S1) and outlet of outlet of the Honghu Lake (S10) were recommended as the priority control areas. Specifically, it is necessary to pay more attention to S1, S4, S5, S6, S9 and S16 when decision making for their calculated membership values (probabilities) of adjacent risk levels quite close.

## 1. Introduction

Heavy metals have adverse effects on aquatic environment, living organisms and human health, due to their toxicity, persistence and bioaccumulation [1,2,3]. Most heavy metals are discharged into water environment by human activities, and then transferred to the sediments gradually via physical, chemical and biological process [4,5]. Furthermore, sediment heavy metals might be released into water again when environmental conditions (such as pH, Eh and salinity) of the sediment–water interface changed [6,7,8]. Thus, heavy metal content in sediments is regarded as a key indicator of aquatic environmental quality. It can reflect the naturally geologic information and the effects caused by intensity and time of anthropogenic activities [9,10]. It is widely recognized that the distribution, mobility and eco-toxicity of heavy metals in sediments depends not only on their total concentration but also on their fraction [11,12,13]. Therefore, it is necessary to take into account heavy metal total content as well as their chemical fractions in sediment [14,15].

Several studies on contamination characterization of heavy metals in sediments focused on the description of the spatial distribution and enrichment degree, the identification of dominant anthropogenic sources by correlation analysis, the research on corresponding bioaccessibility by chemical fraction analysis, and contamination degree or ecological risk assessment using various indexes [16,17,18]. The enrichment factors (EF), contamination factor (CF), sediment quality guidelines (SQGs), and geo-accumulation index (*I_geo_*) were frequently used as indexes of enrichment degree [19,20,21,22]. The potential ecological index (PEI) and risk assessment code (RAC) were widely utilized to identify the ecological risk and bioaccessibility of heavy metals, respectively [16,23]. These existing methods mostly evaluate from single aspect, without considering metal enrichment, bioaccessibility and biotoxicity comprehensively. The PEI assess ecological risk caused by heavy metals from total content and biotoxicity, ignoring effects on bioaccessibility caused by varying metals’ chemical fractions [24]. By contrast, the Risk assessment code (RAC) emphasizes the bioaccessibility of heavy metals based on metals’ chemical fractions, but there is some discrepancy between the bioaccessibility and biotoxicity [25]. Moreover, the above deterministic methods probably lead to biased assessment conclusion or even an unreliable one due to the complexity and fuzziness of environmental assessment system [26,27]. Being the above indexes focused always on heavy metals’ total content or chemical fraction in deterministic assessment system, which may confuse decision makers, it is of significance to explore an assessment method synthetically considering heavy metals’ total content, chemical fraction and systematic uncertainty.

Honghu Lake, which lies in central Hubei Province, is the seventh largest freshwater lake in China. The Ramsar convention included the Honghu Lake as an important wetland nature reserve from 2008, owing to its high ecological value. Besides, the Honghu Lake area is an important agricultural production base, and the environment quality of Honghu Lake has great impact on the health of residents, economic development and social process of surrounding area [28]. Studies showed that the sediments of Honghu Lake were polluted by heavy metals to a certain degree [28,29,30]. Makokha et al. [29] investigated on concentrations, anthropogenic sources and ecological risk of heavy metals (Cr, Ni, Cu, Zn, As, Cd, Hg and Pb) in surface water and sediments of Honghu Lake, and all heavy metals in sediments showed low to moderate ecological risk. Zheng et al. [30] researched on concentrations of heavy metals (Cu, Cd, Zn and Pb) in sediments from Honghu Lake, and *I_geo_* values showed that Cd was at moderate to heavy contamination. There are few studies on chemical fractions of heavy metals in sediments of Honghu Lake. It is essential to take further study on the heavy metal concentrations and their chemical fractions in the sediments of Honghu Lake.

The objectives of this study were: (1) to determine concentrations and spatial distributions of heavy metals (Cr, Cu, Pb, Zn and Cd) in surface sediments from the Honghu Lake; (2) to study characteristics of heavy metals’ chemical fractions; (3) to assess risk of heavy metals by a developed fuzzy comprehensive risk assessment method; and (4) to identify the priority pollutants and areas.

## 2. Materials and Methods

### 2.1. Study Area

**Figure 1:**
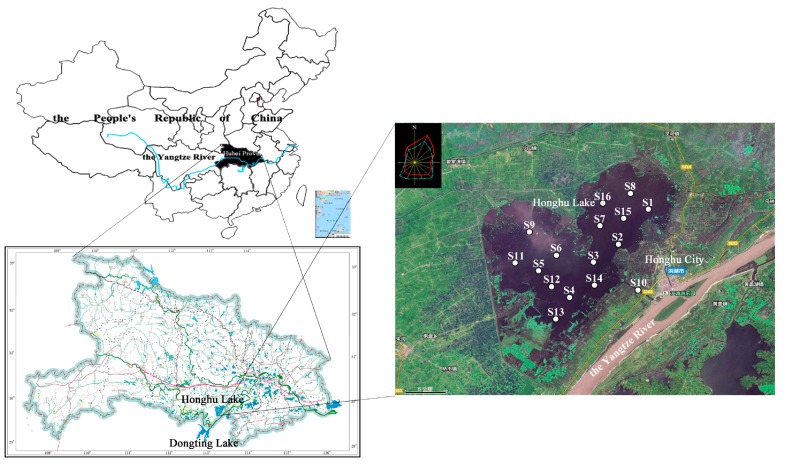
Sediment samples collecting locations in Honghu Lake.

Honghu Lake, the biggest natural wetland in the Hubei Province, lies in Jianghan Plain between the Yangtze River and Han River (Figure 1). The lake has polygonal shape with an area of about 344 km^2^ (29°39′–30°02′ N; 113°07′–114°02′ E). It is 23.4 km from east to west and 20.8 km from north to south with the coastline length of 1354 km. The average depth of the lake is about 1.34 m, with the maximum depth of 2.30 m. Honghu Lake has a north subtropical humid monsoon climate with the annual average temperature of 15.9–16.6 °C, and the annual average rain precipitation is 1000–1300 mm. With rapid development of the local economy, Honghu Lake was polluted by heavy metals to some extent, due to massive discharge of wastewater from industrial, domestic and agricultural sources from Honghu City and Jianli City [28,31,32].

### 2.2. Samples Collection

Surface sediment samples were collected from 16 typical locations (Figure 1) with the help of a global position system (GPS) device in September 2016. The upper 0–10 cm depth of sediments was collected using a mini Beeker type sampler, kept in polytetrafluoroethylene (PTFE) bags and then transferred rapidly to the laboratory in Wuhan. The number of collected samples in each site was 3 and samples in each site were mixed into a composite sample before analyzed. In the laboratory, samples were put evenly on the plastic film to dry naturally in a cool ventilated place. Then, samples were crushed into small pieces by using a pestle and mortar. After that, the samples were sieved through a 10 mesh nylon sieves to remove stones and plant residue. Finally, all samples were processed with another 100 mesh sieve and kept in the plastic bottles prior to analyses.

### 2.3. Analysis Methods

For the determination of total heavy metal content, 0.25 g treated samples were accurately taken by an electronic analytical balance (BSA124S, Goettingen, Germany). Afterwards, the samples were put into digestion vessels and digested with HCl, HNO_3_, HF, and HClO_4_ by the graphite furnace digestion instrument. Then, the solutions were diluted into a final volume of 50 mL with 2% (*v/v*) HNO_3_. The heavy metal contents of Cr, Cu, Pb, Zn and Cd were all detected by Atomic Absorption Spectroscopy (AAS ZEEnit700, Jena, Germany). The distribution of heavy metals in different chemical fractions were determined by optimized BCR three-stage extraction procedure which is widely applied in the studies on heavy metal fraction and the steps of sequential extraction have been provided elsewhere [18,33,34]. According to the types and sequence of extracting agent, the heavy metal fraction in sediments can be divided in to acid-extractable fraction (F1), reducible fraction (F2), oxidizable fraction (F3) and residual fraction (F4) [27,35]. Sediment pH was determined using a glass electrode method (Mettler-Toledo FE20, Zurich, Switzerland) [36]. Sediment organic matter (SOM) was determined by K_2_Cr_2_O_7_ digestion method [37].

To ensure reliability and accuracy of the analysis results, the quality assurance and quality control were strictly assessed using blank samples, parallel samples and standard reference materials (GBW07423). The analysis results were reliable when repeat sample analysis error was below 5%, and the analytical precision for replicate samples was within ±10%. Accepted recoveries of standard samples ranged from 90% to 108%.

### 2.4. Assessment of Sediment Pollution

#### 2.4.1. Geo-Accumulation Index

The geo-accumulation index (*I_geo_*) was proposed by Muller (1969) [38], which was widely used to study heavy metals enrichment in sediment [39,40,41]. It is calculated using the following formula:

(1) $$I_{geo}=\log_{2}(C_{A}^{i}/kC_{0}^{i})$$

where $C_{A}^{i}$ is actually measured concentration of the heavy metal *i* in the sediment samples. *k* is corrected coefficient, which take account variation of background value caused by anthropogenic influences or lithologic variations in the sediments (in general *k* = 1.5) [42,43]. $C_{0}^{i}$ is reference value of heavy metal concentration in sediment. Due to the absence of sediment background values for Honghu Lake, soil background values for Hubei Province were used as a reference, and the values of Cr, Cu, Zn, Pb and Cd were 86.0 mg/kg, 30.7 mg/kg, 83.6 mg/kg, 26.7 mg/kg, and 0.172 mg/kg, respectively (CNEMC 1990) [44].

*I_geo_* is classified into seven levels as follows: (1) *I_geo_* ≤ 0, uncontaminated; (2) 0 < *I_geo_* ≤ 1, uncontaminated to moderately contaminated; (3) 1 < *I_geo_* ≤ 2, moderately contaminated; (4) 2 < *I_geo_* ≤ 3, moderately contaminated to heavily contaminated; (5) 3 < *I_geo_* ≤ 4, heavily contaminated; (6) 4 < *I_geo_* ≤ 5, heavily to extremely contaminated; and (7) *I_geo_* > 5, extremely contaminated.

#### 2.4.2. Potential Ecological Risk Index

The potential ecological risk index (PEI) method, established by Hakanson (1980) [19], is based on the principles of sedimentology. It is widely used by scholars to assess the pollution and potential ecological risk of heavy metal in sediment. This method not only accounts for the content of heavy metals in sediments, but also connects the ecological and environmental effects of heavy metals to environment toxicology [1,3].

(2) $$E_{r}^{i}=T_{r}^{i}\cdot C_{f}^{i}$$

(3) $$C_{f}^{i}=C_{A}^{i}/C_{0}^{i}$$

where $E_{r}^{i}$ is potential risk of individual heavy metal. $T_{r}^{i}$ is toxic-response factor for a given heavy metal, which reflects toxic level and environmental sensitivity of the heavy metal. $C_{f}^{i}$ is contamination factor. $C_{A}^{i}$ is actually measured concentration of the heavy metal in the sediment. $C_{0}^{i}$ is reference value of heavy metal concentration in sediment.

For $T_{r}^{i}$, the Hakanson recommended values of Cr, Cu, Zn, Pb and Cd are 2, 5, 1, 5 and 30, respectively [19]. For $C_{0}^{i}$, soil background values for Hubei Province were used as a reference, and the values of Cr, Cu, Zn, Pb and Cd were 86.0 mg/kg, 30.7 mg/kg, 83.6 mg/kg, 26.7 mg/kg, and 0.172 mg/kg, respectively [44]. Five levels of $E_{r}^{i}$ are defined by Hakanson, as shown in Table 1.

#### 2.4.3. Risk Assessment Code

The risk assessment code (RAC) is a quantitative method to evaluate the degree of heavy metal mobility and bioaccessibility based on metal total concentration and chemical fraction. Since acid-extractable fraction (F1) including exchange fraction and carbonate fraction has higher bioaccessibility, mass fraction of acid-extractable fraction is used to assess the bioaccessibility levels of metals in soil or sediment by this method [45].

(4) $$RAC_{i}=C_{f1}^{i}/C_{A}^{i}$$

where $RAC_{i}$ is risk assessment code index of heavy metal in sediment, $C_{f1}^{i}$ is the concentration of acid-extractable fraction in sediment, and $C_{A}^{i}$ is the actually measured concentration of the heavy metal in sediment. The five levels of $RAC_{i}$ are displayed in Table 2.

#### 2.4.4. Fuzzy Comprehensive Risk Assessment Method

Based on PEI, RAC and fuzzy theory, it is of significance to explore an assessment method synthetically considering heavy metals’ total content, ecological risk, bioaccessibility and fuzziness of assessment system. Therefore, a new fuzzy comprehensive assessment method was developing to identify the comprehensive risk of sediment heavy metals efficiently. Based on the fuzzy comprehensive evaluation theory [46,47], the comprehensive risk was defined as follows:

(5) $$\mathrm{Risk}=f\left(Risk_{A},Risk_{B}\right)$$

where $Risk_{A}$ represents potential ecological risk of heavy metals in sediment, which characterized by $E_{r}^{i}$; $Risk_{B}$ represents bioaccessibility of heavy metals in sediment, which characterized by RAC; and *f* represents the comprehensive risk calculation functions.

Fuzzy language recognition theory in fuzzy mathematic was used to identify the risk in this model. The comprehensive risk can be calculated as follows:

(6) $$\mathrm{Risk}=\tilde{C}\cdot \tilde{R}=\left(C_{1},C_{2}\right)\cdot \left(\begin{array}{c} \begin{array}{cccc} A_{1} & A_{2} & A_{3} & \begin{array}{cc} A_{4} & A_{5} \end{array} \end{array}\\ \begin{array}{cccc} B_{1} & B_{2} & B_{3} & \begin{array}{cc} B_{4} & B_{5} \end{array} \end{array} \end{array}\right)$$

where $\tilde{C}\cdot \tilde{R}$ characterize the *f* in Equation (5). *C* is the weight values of *Risk_A_* and *Risk_B_*. *C_1_* and *C*_2_ were determined as 0.3 and 0.7 by the Delphi method, which indicated that the biotoxicity of heavy metals depend on chemical fractions more than corresponding total contents by expert advice [48,49]. $\tilde{R}$ is membership matrix for levels of *Risk_A_* and *Risk_B_*. *A*_1_, *A*_2_, *A*_3_, *A*_4_, and *A*_5_ represent membership degrees of five levers of *Risk_A_* (Table 1), and *B*_1_, *B*_2_, *B*_3_, *B*_4_, and *B*_5_ represent membership degrees of five levers of *Risk_B_* (Table 2).

Therefore, the comprehensive risk can be represented as a matrix with one row and five columns. The calculated comprehensive risks were divided into five levels as follows: (1) level I, low risk; (2) level II, moderate risk; (3) level III, considerable risk; (4) level IV, high risk; and (5) level V, very high risk. The membership degree of each assessment factor plays a key role in the fuzzy comprehensive risk assessment, which is based on the soil single evaluation indexes and foundation of comprehensive fuzzy assessment. According to Table 1 and Table 2, the membership function of *Risk_A_* and *Risk_B_* was established, and membership degree of each level can be calculated by the following formulas [46,47]:

(1) *Risk_A_*
  (7) $$u_{1}(r)=\left\{ \begin{array}{l} 1,\;r\in \left[0,40\right)\\ (80-r)/40,\;r\in \left[40,80\right)\\ 0,\;r\in \left[80,+\infty \right) \end{array}\right.$$
  (8) $$u_{2}(r)=\left\{ \begin{array}{l} 0,\;r\in \left[0,40\right)\;or\;\left[160,+\infty \right)\\ (r-40)/40,r\in \left[40,80\right)\\ (160-r)/80,r\in \left[80,160\right) \end{array}\right.$$
  (9) $$u_{3}(r)=\left\{ \begin{array}{l} 0,\;r\in \left[0,80\right)\;or\;\left[320,+\infty \right)\\ (r-80)/80,\;r\in \left[80,160\right)\\ (320-r)/160,\;r\in \left[160,320\right) \end{array}\right.$$
  (10) $$u_{4}(r)=\left\{ \begin{array}{l} 0,\;r\in \left[0,160\right)\\ (r-160)/160,\;r\in \left[160,320\right)\\ 0,\;r\in \left[320,+\infty \right) \end{array}\right.$$
  (11) $$u_{5}(r)=\left\{ \begin{array}{l} 0,\;r\in \left[0,320\right)\\ 1,\;r\in \left[320,+\infty \right) \end{array}\right.$$
(2) *Risk_B_*
  (12) $$u_{1}(r)=\left\{ \begin{array}{l} 1,\;r\in \left[0,1\right)\\ (10-r)/9,\;r\in \left[1,10\right)\\ 0,\;r\in \left[10,100\right] \end{array}\right.$$
  (13) $$u_{2}(r)=\left\{ \begin{array}{l} 0,\;r\in \left[0,1\right)or\left[30,100\right]\\ (r-1)/9,\;r\in \left[1,10\right)\\ (30-r)/20,\;r\in \left[10,30\right) \end{array}\right.$$
  (14) $$u_{3}(r)=\left\{ \begin{array}{l} 0,r\in \left[0,10\right)\;or\;\left[50,100\right]\\ (r-10)/20,\;r\in \left[10,30\right)\\ (50-r)/20,\;r\in \left[30,50\right) \end{array}\right.$$
  (15) $$u_{4}(r)=\left\{ \begin{array}{l} 0,\;r\in \left[0,30\right)\\ (r-30)/20,\;r\in \left[30,50\right)\\ (100-r)/50,\;r\in \left[50,100\right] \end{array}\right.$$
  (16) $$u_{5}(r)=\left\{ \begin{array}{l} 0,\;r\in \left[0,50\right)\\ (r-50)/50,\;r\in \left[50,100\right] \end{array}\right.$$

### 2.5. Spatial Analysis

Geographic information system (GIS) was used to present the spatial distribution of heavy metals and risk level in Honghu Lake [21,50]. A proper interpolation method could reduce the corresponding parameter uncertainty and increase the credibility of evaluation. Considering precondition (sampling point distribution, sample numbers and so on) of commonly used methods, the inverse distance weighted method (IDW) was finally adopted to provide spatially detailed information for decision-makers [51,52]. IDW can carry out the spatial analysis for points, which is relatively independent of the surrounding data points, and does not induce the inacceptable interpolation model smoothing effect under relatively limited sample data set [28]. A series of spatial distribution maps was made based on IDW using the ArcGIS 9.3 software (ArcGIS 9.3, Environmental Systems Research Institute Inc., Redlands, CA, USA).

## 3. Results and Discussion

### 3.1. Sediment Characteristics and Mean Concentrations of Heavy Metals

Sediment samples display the pHs between 6.81 and 7.30 with an average value of 7.08, indicating a relative safer environment for heavy metal stabilization. SOM in sediments varied from 3.00 g/kg to 12.91 g/kg with an average value of 7.30 g/kg. Concentrations, *I_geo_* values and sediment quality guidelines (SQGs) are listed in Table 3. Average concentrations of Cu, Zn and Cd exceeded their corresponding background values, while average concentrations of Cr and Pb were lower than the background values. In particular, the average measured value of Cd was 2.5 times than its background value. SQG is established to evaluate the toxicity or risk of contaminants to aquatic ecosystems [52,53]. When the heavy metal’ concentration is lower than the threshold effect level (TEL), it means that adverse biological toxicity effects rarely occur. When concentration is higher than the probable effect level (PEL), it means that adverse biological toxicity effects frequently occur [52,53]. Compared with the corresponding TELs, the enrichment degree of the studied five metals in sediment decreased in the sequence of Cr > Cu > Zn > Pb > Cd. Generally, the concentrations of all heavy metals in 16 sampling sites were lower than Grade II values of the Chinese Environmental Quality Standard for Soils (GB 15618-1995). According to the calculated results of *I_geo_* (Figure S1), it is obvious that Cd in all sampling sites were under un-contamination to moderate contamination, while sediments was not contaminated by Cr, Cu, Pb and Zn for their *I_geo_* less than 0. Specifically, Cd in S14 has the highest *I_geo_* value and Cd in S13 and S14 belonged to moderately contamination. Average *I_geo_* values of five kinds of heavy metals decreased in the order of Cd > Zn > Cu > Pb > Cr. 

### 3.2. Spatial Distribution of Heavy Metals in Surface Sediments from the Honghu Lake

**Figure 2:**
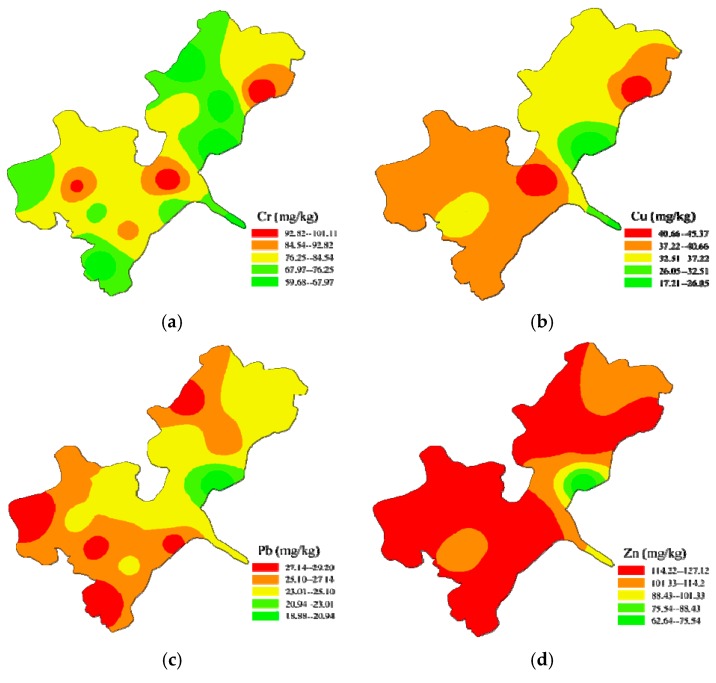

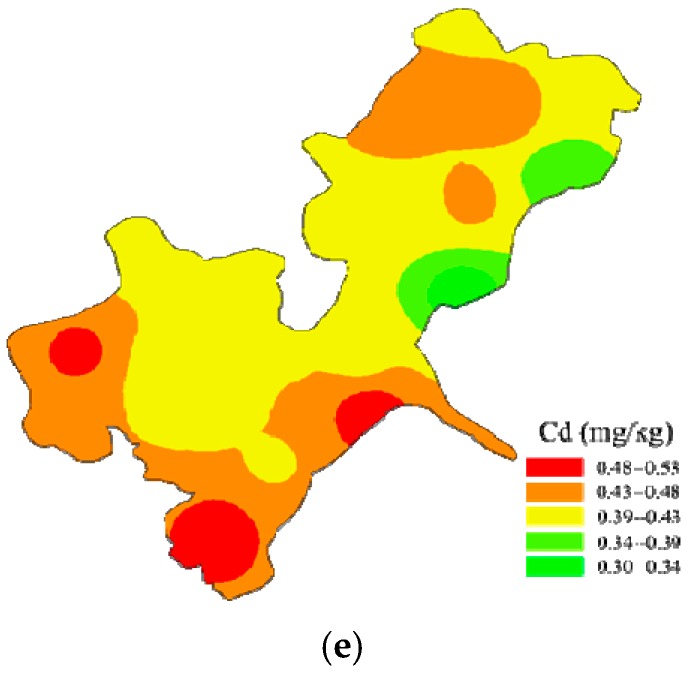
Spatial distributions of: Cr (**a**); Cu (**b**); Pb (**c**); Zn (**d**); and Cd (**e**) in surface sediments from Honghu Lake.

Figure 2 shows the spatial distribution of Cr, Cu, Pb, Zn and Cd in sediments throughout Honghu Lake. The detailed concentration values of these metals are shown in Figure S2. To characterize spatial distribution of heavy metals effectively, Honghu Lake was divided into three regions: North Honghu Lake (S1, S2, S7, S8, S15 and S16), South Honghu Lake (S3, S4, S5, S6, S9, S11, S12, S13 and S14), and the outlet of Honghu Lake (S10).

According to Figure 2a,b, there were similar spatial distributions of relatively higher pollution points for Cr and Cu. The enrichment degrees of Cr and Cu in South Honghu Lake and the east part of North Honghu Lake were higher than the other parts. Concentrations of Cr varied from 59.68 mg/kg (S15) to 101.11 mg/kg (S3). Unlike other heavy metals, only concentrations of Cr (in S1, S3, and S5) exceeded the corresponding PEL, which indicates that adverse biological toxicity effects may frequently occur in these areas. For Cu, the lowest and highest concentrations were found in S2 (17.21 mg/kg) and in S3 (45.37 mg/kg). Based on Figure 2b, for Cu, 25% of sample concentrations were lower than the TEL (35.7 mg/kg).

For Pb, the highest concentration of 29.20 mg/kg was found in S11 and the lowest concentration of 18.88 mg/kg was found in S2. Based on Figure 2c, 37.5% of samples of Pb exceeded the background value (30.7 mg/kg), while all of the samples did not exceed the TEL (91.3 mg/kg). The lower part of South Honghu Lake and the west part of North Honghu Lake had relatively higher Pb enrichment degree than the other parts.

Concentrations of Zn varied from 62.64 mg/kg (S2) to 127.12 mg/kg (S15). According to Figure 2d, 97.75% of samples exceeded the background value (83.6 mg/kg), while only 18.75% of samples exceeded the TEL. Most parts of Honghu Lake were enriched to some t degree except the small portion of south part of North Honghu Lake.

Concentrations of Cd varied from 0.30 mg/kg (S2) to 0.53 mg/kg (S14). Based on Figure 2e, the contents of Cd in all sampling sites exceeded the corresponding background value (0.172 mg/kg), while no samples exceed the TEL (0.596 mg/kg). In addition, the enrichment degree of Cd in lower part of South Honghu Lake, the west part of North Honghu Lake and the outlet of Honghu Lake were relatively higher than the other parts.

### 3.3. Heavy Metal Chemical Fractions

**Figure 3:**
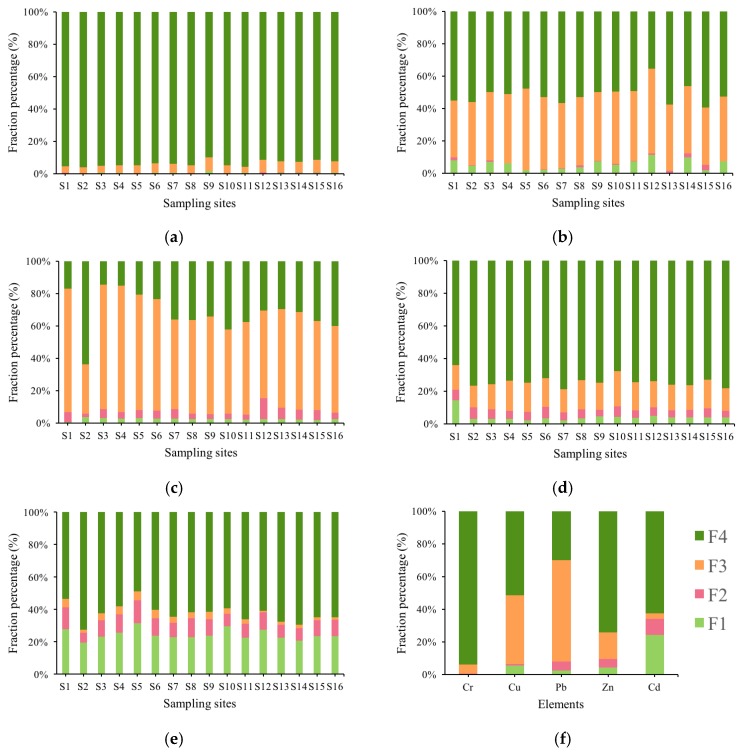
Heavy metal chemical fraction percentage of: Cr (**a**); Cu (**b**); Pb (**c**); Zn (**d**); Cd (**e**); and (**f**) all elements summary (F1: acid-extractable fraction; F2: reducible fraction; F3: oxidizable fraction; F4: residual fraction).

The proportion of heavy metals chemical fractions of Cr, Cu, Pb, Zn and Cr are displayed in Figure 3. The proportions of the acid-extractable fraction decreased in the order of Cd > Cu > Zn > Pb > Cr. Figure 3 shows that the bioaccessibility of Cr is at a low level since no more than 2% of Cr occurred in acid-extractable fraction and reducible fraction. Cr mainly existed in residual fraction, ranging from 89.92% to 96.29%, and Cr in oxidizable fraction ranged from 3.46% to 8.52%. Unlike Cr, Cu in sediments was mainly associated to oxidizable fraction and residual fraction, which ranged from 35.19% to 52.30% and from 35.47% to 59.33%, respectively. The proportions of Cu existing in acid-extractable and reducible fractions ranged from 0.34% to 11.34% and from 0% to 3.37%, respectively.

Different from the other metals, Pb mainly existed in oxidizable fraction in all sampling sites except S2, ranging from 53.12% to 80.53%. The oxidizable fraction of heavy metal is produced by activities of aquatic organisms and discharge of organic wastewater, which is relatively stable in sediment. However, heavy metals existing in oxidizable fraction would become higher valence metals with migration in the strong oxidizing conditions. It means that Pb in sediment from Honghu Lake potentially had some ecological risk.

Zn existed mainly in residual fraction, ranging from 64.18% to 78.83%. The amounts of Zn in acid-extractable fraction, reducible fraction, and oxidizable fraction were in the range from 2.00% to 14.31%, from 3.86% to 6.96% and from 13.35% to 21.56%, respectively.

Unlike other metals, Cd had the higher proportion of acid-extractable fraction (19.41–31.47%) while mainly occurred in residual fraction (53.57–72.55%). The acid-extractable fraction is very sensitive to water environment change, which be probably released in acidic or neutral condition and easily transfer into aquatic organisms. It showed that Cd in sediment from sampling sites might have a relatively higher biological toxicity than the other metals.

### 3.4. Fuzzy Comprehensive Risk Assessment

According to the analysis above, results were obtained by different evaluation indexes such as SQGs, PEI and RAC. The enrichment degree compared with SQGs decreased in the order of Cr > Cu > Zn > Pb > Cd, average ecological risks were decreased in the order of Cd > Cu > Pb > Cr > Zn, and the average bioaccessibilities decreased in the order of Cd > Cu > Zn > Pb > Cr. Results showed that some differences surely existed among these widely used methods, which may confuse decision makers because these methods unilaterally focus on ecological risk based on total content or bioaccessibility based on chemical fraction. Moreover, there are complexity and fuzziness in environmental assessment system, which need to be under quantitative reduce and control. Therefore, a new model with synthetically considering heavy metals’ total content, ecological risk, bioaccessibility and systematic fuzziness is needed. To identify the comprehensive risk of sediment heavy metals efficiently, the fuzzy comprehensive assessment method was established based on PEI, RAC and fuzzy theory. To make the assessment method scientific, rational and high recognition, firstly, potential ecological risk and bioavailability were divided into different levels using fuzzy mathematics, then fuzzy results of PEI and RAC were endowed with weights, and finally comprehensive risk can be calculated as in Equations (5) to (16). Combining Table 1 and Table 2, according to arithmetic calculation from Equations (1) to (16), an assessment matrix based on average values of Cr, Cu, Pb, Zn and Cd is as follows:

$$R_{Cr}=\left(\begin{array}{c} \begin{array}{cccc} 1 & 0 & 0 & \begin{array}{cc} 0 & 0 \end{array} \end{array}\\ \begin{array}{cccc} 1 & 0 & 0 & \begin{array}{cc} 0 & 0 \end{array} \end{array} \end{array}\right),\;R_{Cu}=\left(\begin{array}{ccccc} 1 & 0 & 0 & 0 & 0\\ 0.507 & 0.493 & 0 & 0 & 0 \end{array}\right),$$

$$R_{Pb}=\left(\begin{array}{ccccc} 1 & 0 & 0 & 0 & 0\\ 0.831 & 0.169. & 0 & 0 & 0 \end{array}\right),\;R_{Zn}=\left(\begin{array}{ccccc} 1 & 0 & 0 & 0 & 0\\ 0.636 & 0.364 & 0 & 0 & 0 \end{array}\right),$$

$$R_{Cd}=\left(\begin{array}{ccccc} 0.106 & 0.894 & 0 & 0 & 0\\ 0 & 0.282 & 0.718 & 0 & 0 \end{array}\right)$$

Based on Equation (6), average comprehensive risks of heavy metals in sediments of Honghu Lake were as follows: *Risk_Cr_* = (1, 0, 0, 0, 0), *Risk_Cu_* = (0.655, 0.345, 0, 0, 0), *Risk_Pb_* = (0.882, 0.118, 0, 0, 0), *Risk_Zn_* = (0.745, 0.255, 0, 0, 0) and *Risk_Cd_* = (0.032, 0.466, 0.502, 0, 0). According to maximum membership principle, average comprehensive risks of heavy metals decreased in the sequence of Cd (considerable risk) > Cu (moderate risk) > Zn (low risk) > Pb > Cr. It indicated that Cd and Cu were likely to have adverse biological effects on aquatic systems of the Honghu Lake.

Unlike other metals, the comprehensive risk of Cd belonged to the considerable risk level, which is because Cd had a moderate ecologic risk and a high level of bioaccessibility. Since the membership values of moderate risk level and considerable risk level were very close and the results is greatly affect by subjective factors, further monitoring and expert discussion about the risk level of Cd is necessary when decision making. Although the bioaccessibility of Cu was close to the moderate level, its comprehensive risk was at low risk level because of its low ecological risk. The membership degree of low risk level (0.507) and moderate risk level (0.493) was also close. Under principle of maximum risk protection, Cu was determined as moderate risk. If the absolute difference between memberships of two risk levels is less than 10%, the final risk level can be determined as the higher level for warning. Average comprehensive risk of Cr in sediments belonged to the low risk level because of its very low ecological risk and quite low biological risk. For Cr, Zn and Pb, they were also at the low comprehensive risk level due to their low ecological risk and low biological risk.

**Figure 4:**
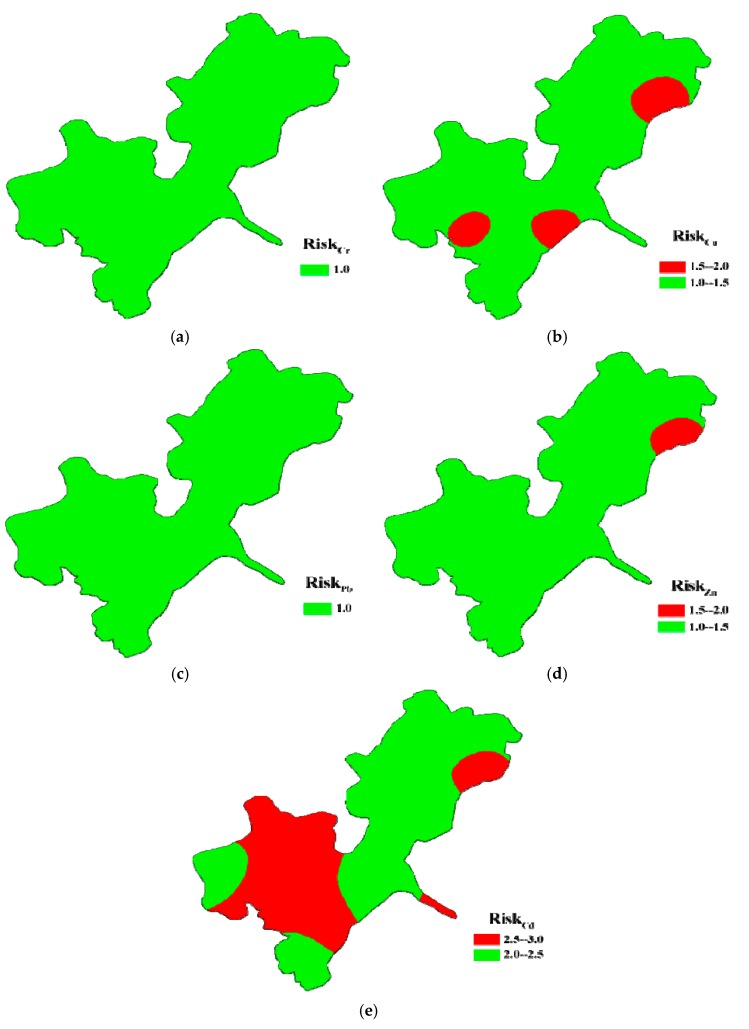
Comprehensive risk mapping of: Cr (**a**); Cu (**b**); Pb (**c**); Zn (**d)**; and Cd (**e**) in surface sediments from Honghu Lake.

**Figure 5:**
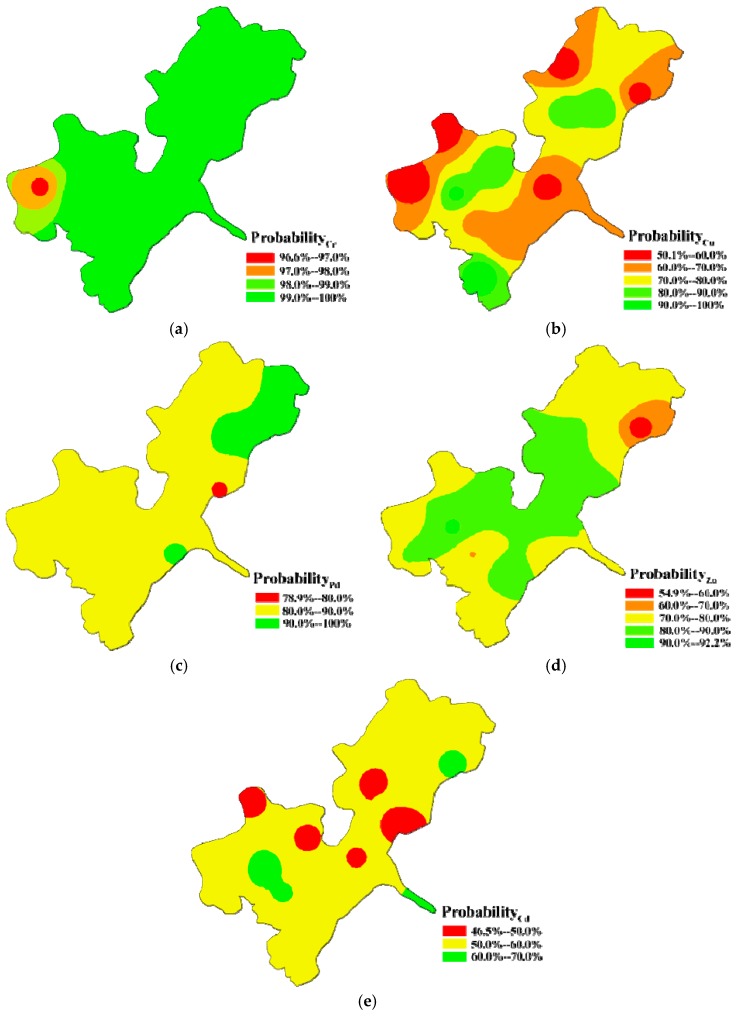
Probability subjection mapping of comprehensive risk of: Cr (**a**); Cu (**b**); Pb (**c**); Zn (**d**); and Cd (**e**).

Spatial distributions of the calculated comprehensive risk levels and their probabilities for heavy metals in each sediment sample from Honghu Lake are shown in Figure 4 and Figure 5, which were drawn according to the results listed in Table S1. Compared with other metals, Cd in sediments was at the highest risk level varying from moderate risk to considerable risk. The areas under considerable risk were about 43.75% of Honghu Lake, with 57.14% samples belonging to considerable risk with about 50% membership degree (Figure 4e and Figure 5e). The central part of South Honghu Lake (S4, S5, S6, S9 and S12), east part of North Honghu Lake (S1) and outlet of outlet of Honghu Lake (S10) were the priority control areas. Moreover, sites including S2, S3, S4, S5, S6, S7, S8, S9, S11, S12, S13, S14, S15 and S15 need to be paid more attention when decision making because the probabilities of their considerable risk level was 40–50%. Spatially, the comprehensive risks of Cd decreased in the order of S5 > S10 > S1 > S12 > S4 > S9 > S6 > S15 > S13 > S16 > S11 > S3 > S8 > S7 > S14 > S2. Moreover, risk levels of Cd were not completely determined in S3, S6, S9, S11, S13, S16 and S12 because their calculated membership values of moderate risk level and considerable risk level were quite close.

According to Figure 4b, for Cu, there were 81.25% and 18.75% of areas under low risk and moderate risk, respectively. The areas around S1, S12 and S14 of South Honghu Lake were of concern. Specifically, the membership degrees in S1, S3, S9, S11 and S16 were around 50% (Figure 5b). It is necessary to pay more attention to these sites during corresponding decision making. The comprehensive risks of Cu were in the descending order of S12 > S14 > S1 > S11 > S16 > S9 > S3 > S4 > S10 > S2 > S8 > S7 > S6 > S15 > S5 > S13.

Comparing with Cd and Cu, the comprehensive risks of Cr, Pb and Zn were at lower level. Comprehensive risks of Cr and Zn in sediments from 16 sampling sites were all at low risk level. Calculated risk values of Cr in samples except S9 were extremely close, and S9 was slightly higher than other sampling sites. Comprehensive risks of Pb decreased in the order of S2 > S5 > S3 > S4 > S6 > S7 > S10 > S8 > S13 > S9 > S12 > S16 > S14 > S15 > S1. Comprehensive risks of Zn in sediment at all sampling sites except S1 (belonging to moderate risk level) were at low risk level. Calculated comprehensive risks of Zn decreased in the order of S1 > S12 > S9 > S10 > S13 > S14 > S16 > S15 > S11 > S8 > S6 > S2 > S3 > S4 > S5 > S7.

## 4. Conclusions

Average *I_geo_* values of the studied heavy metals decreased in the order of Cd > Zn > Cu > Pb > Cr. From metal chemical fractions’ proportions, Cr, Cu, Zn and Cd were dominated by their residual fraction while Pb was dominated by its oxidizable fraction. Cd had the highest proportion of acid-extractable fraction, which reached 24.35%. Compared with the corresponding TELs, adverse effects of studied five metals in sediments decreased in the sequence of Cr > Cu > Zn > Pb > Cd. Average potential ecological risk indexes were the descending order: Cd, Cu, Pb, Cr, Zn. Moreover, average RAC values were ranked in the order: Cd > Cu > Zn > Pb > Cr. Different results by different indexes probably confuse or even mislead decision-makers. Thus, the fuzzy comprehensive risk assessment model was established. Average comprehensive risks of heavy metals decreased in the order of Cd (considerable risk) > Cu (moderate risk) > Zn (low risk) > Pb > Cr. Cd and Cu were determined as the contaminants of most concern among the five metals, which differed to a certain extent from results of *I_geo_* and SQGs method. For Cd, the central part of South Honghu Lake (S4, S5, S6, S9 and S12), east part of North Honghu Lake (S1) and outlet of outlet of Honghu Lake (S10) were the priority control areas. Notice that 57.14% Cd samples, which belonged to considerable risk, need to be paid more attention for their 40–50% membership degrees. For Cu, the areas around S1, S12 and S14 of South Honghu Lake were of concern, and area around S1 needs to be paid more attention by decision makers for their final risk judgment. Overall, the developed comprehensive risk assessment method proved to be an improvement on previous methods in as it synthetically incorporated the heavy metals’ total content, ecological risk, bioaccessibility and systematic parameter uncertainty control to provide not only a comprehensive risk level but also its membership degree, which reflects the probabilities of the risk level. Multivariate statistical analysis among heavy metals, pH and SOM need to be under further investigation to identify the contributions of the anthropogenic pollution such as urban lives, industries, and agriculture. It is advisable to develop environmental management strategies for the identified the contaminants and areas of most concern for further protecting and improving the aquatic environment in Honghu Lake.

## Figures and Tables

**Table 1:** Ecological risk levels of a single metal pollution [19].

Level	$E_{r}^{i}$ Value	Extent of Ecological Risk of Single Metal
I	$E_{r}^{i}$ < 40	Low potential ecological risk
II	40 ≤ $E_{r}^{i}$ < 80	Moderate ecological risk
III	80 ≤ $E_{r}^{i}$ < 160	Considerable ecological risk
IV	160 ≤ $E_{r}^{i}$ < 320	High ecological risk
V	$E_{r}^{i}$ ≥ 320	Very high ecological risk

**Table 2:** Risk levels of heavy metals based on risk assessment code (RAC).

Level	$RAC_{i}$ Value	Risk Degree
I	$RAC_{i}$ < 1	No biological risk
II	1 ≤ $RAC_{i}$ < 10	Low biological risk
III	10 ≤ $RAC_{i}$ < 30	Moderate biological risk
IV	30 ≤ $RAC_{i}$ < 50	High biological risk
V	50 ≤ $RAC_{i}$ ≤ 100	Very high biological risk

**Table 3:** Statistics of heavy metal concentrations in sediments from Honghu Lake (mg/kg).

Elements	Cr	Cu	Pb	Zn	Cd
Min	59.68	17.21	18.88	62.64	0.30
Max	101.11	45.37	29.20	127.12	0.53
Mean	76.36	36.41	25.29	113.79	0.43
S.D. ^1^	14.16	6.89	2.90	16.38	0.06
Background ^2^	86.0	30.7	26.7	83.6	0.172
Grade II ^3^	300	100	80	250	0.5
*I_geo_*	−0.75	−0.34	−0.66	−0.14	0.77
TEL ^4^	37.3	35.7	35	123.1	0.596
PEL ^5^	90	196.6	91.3	314.8	3.53

^1^ S.D.: standard deviation; ^2^ Background: soil background values of Hubei province (CNEMC 1990); ^3^ Grade II: The Grade II standard values of the Chinese Environmental Quality Standard for Soils (GB 15618-1995); ^4^ TEL: the threshold effect level [53]; ^5^ PEL: the probable effect concentration [53].

## Supplementary Materials

The following are available online at http://www.mdpi.com/1660-4601/15/2/207/s1, Figure S1: *I_geo_* values of five heavy metals from each sampling site. Figure S2: Concentrations of heavy metals in 16 sampling sites. Table S1: The comprehensive risks of Cr, Cu, Pb, Zn and Cd in sediments from Honghu Lake.

## References

[1] Li F., Huang J.H., Zeng G.M., Yuan X.Z., Li X.D., Liang J., Wang X.Y., Tang X.J., Bai B. (2013). Spatial risk assessment and sources identification of heavy metals in surface sediments from the Dongting Lake, Middle China. J. Geochem. Explor..

[2] Liu J.Y., Liang J., Yuan X.Z., Zeng G.M., Yuan Y.J., Wu H.P., Huang X.L., Liu J.F., Hua S.S., Li F. (2015). An integrated model for assessing heavy metal exposure risk to migratory birds in wetland ecosystem: A case study in Dongting Lake Wetland, China. Chemosphere.

[3] Lin Q., Liu E.F., Zhang E.L., Li K., Shen J. (2016). Spatial distribution, contamination and ecological risk assessment of heavy metals in surface sediments of Erhai Lake, a large eutrophic plateau lake in southwest China. Catena.

[4] Guo W., Huo S.L., Xi B.D., Zhang J.T., Wu F.C. (2015). Heavy metal contamination in sediments from typical lakes in the five geographic regions of China: Distribution, bioaccessibility, and risk. Ecol. Eng..

[5] Zahra A., Hashmi M.Z., Malik R.N., Ahmed Z. (2014). Enrichment and geo-accumulation of heavy metals and risk assessment of sediments of the Kurang Nallah—Feeding tributary of the Rawal Lake Reservoir, Pakistan. Sci. Total Environ..

[6] Chen M.S., Ding S.M., Zhang L.P., Li Y.Y., Sun Q., Zhang C.S. (2017). An investigation of the effects of elevated phosphorus in water on the release of heavy metals in sediments at a high resolution. Sci. Total Environ..

[7] Wang L.L., Yuan X.Z., Zhong H., Wang H., Wu Z.B., Chen X.H., Zeng G.M. (2014). Release behavior of heavy metals during treatment of dredged sediment by microwave-assisted hydrogen peroxide oxidation. Chem. Eng. J..

[8] Wen J., Yi Y.J., Zeng G.M. (2016). Effects of modified zeolite on the removal and stabilization of heavy metals in contaminated lake sediment using BCR sequential extraction. J. Environ. Manag..

[9] Li F., Huang J.H., Zeng G.M., Huang X.L., Li X.D., Liang J., Wu H.P., Wang X.Y., Bai B. (2014). Integrated Source Apportionment, Screening Risk Assessment, and Risk Mapping of Heavy Metals in Surface Sediments: A Case Study of the Dongting Lake, Middle China. Hum. Ecol. Risk Assess..

[10] Wang Y.Q., Yang L.Y., Kong L.H., Liu E.F., Wang L.F., Zhu J.R. (2015). Spatial distribution, ecological risk assessment and source identification for heavy metals in surface sediments from Dongping Lake, Shandong, East China. Catena.

[11] Huang J.H., Li F., Zeng G.M., Liu W.C., Huang X.L., Xiao Z.H., Wu H.P., Gu Y.L., Li X., He X.X. (2016). Integrating hierarchical bioaccessibility and population distribution into potential eco-risk assessment of heavy metals in road dust: A case study in Xiandao District, Changsha city, China. Sci. Total Environ..

[12] Luo X.S., Ding J., Xu B., Wang Y.J., Li H.B., Yu S. (2012). Incorporating bioaccessibility into human health risk assessments of heavy metals in urban park soils. Sci. Total. Environ..

[13] Alonso Castillo M.L., Sánchez Trujillo I., Vereda Alonso E., García de Torres A., Cano Pavón J.M. (2013). Bioaccessibility of heavy metals in water and sediments from a typical Mediterranean Bay (Málaga Bay, Region of Andalucía, Southern Spain). Mar. Pollut. Bull..

[14] Xiao Z.H., Yuan X.Z., Li H., Jiang L., Leng L., Chen X., Zeng G.M., Li F., Cao L. (2015). Chemical speciation, mobility and phyto-accessibility of heavy metals in fly ash and slag from combustion of pelletized municipal sewage sludge. Sci. Total. Environ..

[15] Peijnenburg W.J.G.M., Zablotskaja M., Vijver M.G. (2007). Monitoring metals in terrestrial environments within a bioaccessibility framework and a focus on soil extraction. Ecotoxicol. Environ. Saf..

[16] Huang Y., Li Y.X., Gao F.W., Xu M.M., Sun B., Wang N., Yang J. (2015). Speciation and Risk Assessment of Heavy Metals in Surface Sediments from the Heavily Polluted Area of Xiaoqing River. Environ. Sci..

[17] Fu J., Zhao C.P., Luo Y.P., Liu C.S., Kyzas G.Z., Luo Y., Zhao D.Y., An S.Q., Zhu H.L. (2014). Heavy metals in surface sediments of the Jialu River, China: Their relations to environmental factors. J. Hazard. Mater..

[18] Ma X.L., Zuo H., Tian M.J., Zhang L.Y., Meng J., Zhou X.N., Min N., Chang X.Y., Liu Y. (2016). Assessment of heavy metals contamination in sediments from three adjacent regions of the Yellow River using metal chemical fractions and multivariate analysis techniques. Chemosphere.

[19] Hakanson L. (1980). An ecological risk index for aquatic pollution control: A sedimentological approach. Water Res..

[20] Sakan S.M., Đorđević D.S., Manojlović D.D., Polić P.S. (2009). Assessment of heavy metal pollutants accumulation in the Tisza river sediments. J. Environ. Manag..

[21] Li F., Zhang J.D., Huang J.H., Huang D.W., Yang J., Song Y.W., Zeng G.M. (2016). Heavy metals in road dust from Xiandao District, Changsha City, China: Characteristics, health risk assessment, and integrated source identification. Environ. Sci. Pollut. Res..

[22] Xu F.J., Liu Z.Q., Cao Y.C., Qiu L.W., Feng J.W., Xu F., Tian X. (2017). Assessment of heavy metal contamination in urban river sediments in the Jiaozhou Bay catchment, Qingdao, China. Catena.

[23] Maanan M., Saddik M., Maanan M., Chaibi M., Assobhei O., Zourarah B. (2015). Environmental and ecological risk assessment of heavy metals in sediments of Nador lagoon, Morocco. Ecol. Indic..

[24] Zhu H.N., Yuan X.Z., Zeng G.M., Jiang M., Liang J., Zhang C. (2012). Ecological risk assessment of heavy metals in sediments of Xiawan Port based on modified potential ecological risk index. Trans. Nonferrous Met. Soc..

[25] Zhang L., Liao Q.J.H., Shao S.G., Zhang N., Shen Q.S., Liu C., Lin Y.P. (2015). Heavy Metal Pollution, Fractionation, and Potential Ecological Risks in Sediments from Lake Chaohu (Eastern China) and the Surrounding Rivers. Int. J. Environ. Res. Public Health.

[26] Hu B.B., Liu B.Q., Zhou J., Guo J.T., Sun Z.B., Meng W.Q., Guo X., Duan J.H. (2016). Health risk assessment on heavy metals in urban street dust of Tianjin based on trapezoidal fuzzy numbers. Hum. Ecol. Risk Assess..

[27] Li L., Xu Z.R., Zhang C., Bao J., Dai X. (2012). Quantitative evaluation of heavy metals in solid residues from sub- and super-critical water gasification of sewage sludge. Bioresour. Technol..

[28] Li F., Qiu Z.Z., Zhang J.D., Liu C.Y., Cai Y., Xiao M.S. (2017). Spatial distribution and fuzzy health risk assessment of trace elements in surface water from Honghu Lake. Int. J. Environ. Res. Public Health.

[29] Makokha V.A., Qi Y.L., Shen Y., Wang J. (2016). Concentrations, Distribution, and Ecological Risk Assessment of Heavy Metals in the East Dongting and Honghu Lake, China. Expo. Health.

[30] Zheng H., Yang D., Xing X.L., Xing X.L., Zhang Z.Z., Shu Q.L. (2016). Historical records, distribution characteristics and sources of heavy metals from sediment core in Honghu Lake, China. China Environ. Sci..

[31] Hu Y., Qi S.H., Wu C.X., Ke Y.P., Chen J., Chen W., Gong X.Y. (2012). Preliminary assessment of heavy metal contamination in surface water and sediments from Honghu Lake, East Central China. Front. Earth Sci..

[32] Mali M., Dell’Anna M.M., Notarnicola M., Damiani L., Mastrorilli P. (2017). Combining chemometric tools for assessing hazard sources and factors acting simultaneously in contaminated areas. case study: “Mar piccolo” taranto (south Italy). Chemosphere.

[33] Mossop K.F., Davidson C.M. (2003). Comparison of original and modified BCR sequential extraction procedures for the fractionation of copper, iron, lead, manganese and zinc in soils and sediments. Anal. Chimica Acta.

[34] Davidson C.M., Thomas R.P., Mcvey S.E., Perala R., Littlejohn D., Ure A.M. (1994). Evaluation of a sequential extraction procedure for the speciation of heavy metals in sediments. Anal. Chimica Acta.

[35] Chen M., Li X.M., Yang Q., Zeng G.M., Zhang Y., Liao D.X., Liu J.J., Hu J.M., Guo L. (2008). Total concentrations and speciation of heavy metals in municipal sludge from Changsha, Zhuzhou and Xiangtan in middle-south region of China. J. Hazard. Mater..

[36] Ministry of Agriculture of the PRC (2007). NY/T 1377-2007 Determination of pH in Soil.

[37] Page A.L., Miller R.H., Keeney D.R. (1982). Total Carbon, Organic Matter and Carbon, Methods of Soil Analysis Part 2.

[38] Muller G. (1969). Index of geoaccumulation in sediments of the Rhine River. GeoJournal.

[39] Tang R.L., Ma K.M., Zhang Y.X., Mao Q.Z. (2013). The spatial characteristics and pollution levels of metals in urban road dust of Beijing, China. Appl. Geochem..

[40] Li F., Zhang J.D., Jiang W., Liu C.Y., Zhang Z.M., Zhang C.D., Zeng G.M. (2017). Spatial health risk assessment and hierarchical risk management for mercury in soils from a typical contaminated site, China. Environ. Geochem. Health.

[41] Li F., Zhang J.D., Yang J., Liu C.Y., Zeng G.M. (2016). Site-specific risk assessment and integrated management decision-making: A case study of a typical heavy metal contaminated site, Middle China. Hum. Ecol. Risk Assess..

[42] Han X., Lu X., Qing G.L.T., Wu Y. (2017). Health risks and contamination levels of heavy metals in dusts from parks and squares of an industrial city in semi-arid area of china. Int. J. Environ. Res. Public Healt.

[43] Lu X.W., Li L.Y., Wang L.J., Lei K., Huang J., Zhai Y. (2009). Contamination assessment of mercury and arsenic in roadway dust from Baoji, China. Atmos. Environ..

[44] China National Environmental Monitoring Center (CNEMC) (1990). Background Values of Soil Elements in China.

[45] Ke X., Gui S., Huang H., Zhang H., Wang C., Guo W. (2017). Ecological risk assessment and source identification for heavy metals in surface sediment from the Liaohe River protected area, China. Chemosphere.

[46] Zhang Q.W., Yang X.H., Zhang Y., Zhong M. (2013). Risk assessment of groundwater contamination: A multilevel fuzzy comprehensive evaluation approach based on drastic model. Sci. World J..

[47] Liu Y.L., Huang X.L., Duan J., Zhang H.M. (2017). The assessment of traffic accident risk based on grey relational analysis and fuzzy comprehensive evaluation method. Nat. Hazards.

[48] Linstone H.A., Turoff M. (1975). The Delphi Method: Techniques and Applications.

[49] Steurer J. (2011). The delphi method: An efficient procedure to generate knowledge. Skelet. Radiol..

[50] Tang G.A., Yang X. (2016). Geographic Information System Spatial Analysis Experiment Tutorial.

[51] Huang J.H., Liu W.C., Zeng G.M., Li F., Huang X.L., Gu Y.L., Shi L.X., Shi Y.H., Wan J. (2016). An exploration of spatial human health risk assessment of soil toxic metals under different land uses using sequential indicator simulation. Ecotoxicol. Environ. Saf..

[52] Canadian Council of Ministers of the Environment (CCME) (1995). Protocol for the Derivation of Canadian Sediment Quality Guidelines for the Protection of Aquatic Life.

[53] Smith S.L., Macdonald D.D., Keenleyside K.A., Ingersoll C.G., Field L.J. (1996). A preliminary evaluation of sediment quality assessment values for freshwater ecosystems. J. Gt. Lakes Res..

