# Supplementary material for: Spatial Distribution, Chemical Fraction and Fuzzy Comprehensive Risk Assessment of Heavy Metals in Surface Sediments from the Honghu Lake, China
Source: Int J Environ Res Public Health. 2018 Jan 26;15(2):207. doi: 10.3390/ijerph15020207 (PMC5858276; doi:10.3390/ijerph15020207)
Supplement: Supplementary file 1 [file ijerph-15-00207-s001.pdf]

Supplementary Materials

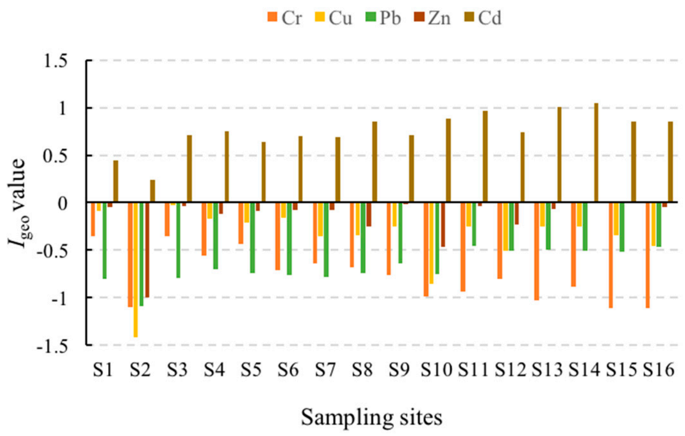

Figure S1. I_{geo} values of five heavy metals from each sampling site.

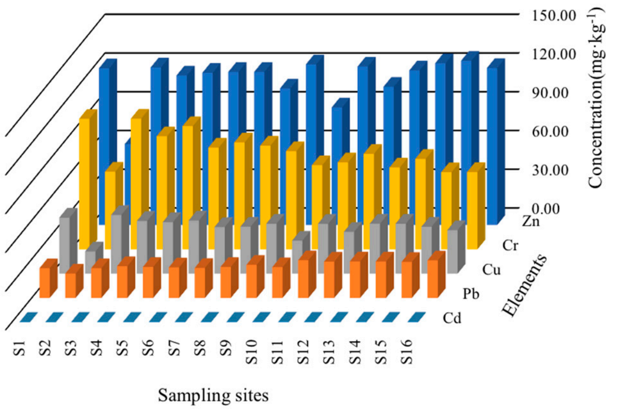

Figure S2. Concentrations of heavy metals in 16 sampling sites.

Table S1. The comprehensive risks of Cr, Cu, Pb, Zn and Cd in sediments from the Honghu Lake

Sampling Sites	Elements				
	Cr	**Cu**	**Pb**	**Zn**	**Cd**
S1	(1, 0, 0, 0, 0)	(0.452, 0.548, 0, 0, 0)	(1, 0, 0, 0, 0)	(0.300, 0.549, 0.151, 0, 0)	(0.147, 0.227, 0.626, 0, 0)
S2	(1, 0, 0, 0, 0)	(0.711, 0.289, 0, 0, 0)	(0.789, 0.211, 0, 0, 0)	(0.833, 0.167, 0, 0, 0)	(0.205, 0.465, 0.330, 0, 0)
S3	(1, 0, 0, 0, 0)	(0.521, 0.479, 0, 0, 0)	(0.840, 0.160, 0, 0, 0)	(0.839, 0.161, 0, 0, 0)	(0.055, 0.485, 0.460, 0, 0)
S4	(1, 0, 0, 0, 0)	(0.601, 0.399, 0, 0, 0)	(0.848, 0.152, 0, 0, 0)	(0.842, 0.158, 0, 0, 0)	(0.037, 0.416, 0.547, 0, 0)
S5	(1, 0, 0, 0, 0)	(0.940, 0.060, 0, 0, 0)	(0.831, 0.169, 0, 0, 0)	(0.922, 0.078, 0, 0, 0)	(0.082, 0.218, 0.649, 0.051, 0)
S6	(1, 0, 0, 0, 0)	(0.915, 0.085, 0, 0, 0)	(0.854, 0.146, 0, 0, 0)	(0.817, 0.183, 0, 0, 0)	(0.058, 0.461, 0.481, 0, 0)
S7	(1, 0, 0, 0, 0)	(0.866, 0.134, 0, 0, 0)	(0.856, 0.144, 0, 0, 0)	(0.922, 0.078, 0, 0, 0)	(0.064, 0.488, 0.448, 0, 0)
S8	(1, 0, 0, 0, 0)	(0.780, 0.220, 0, 0, 0)	(0.877, 0.123, 0, 0, 0)	(0.806, 0.194, 0, 0, 0)	(0, 0.550, 0.450, 0, 0)
S9	(0.966, 0.034, 0, 0, 0)	(0.514, 0.486, 0, 0, 0)	(0.883, 0.117, 0, 0, 0)	(0.733, 0.267, 0, 0, 0)	(0.055, 0.461, 0.484, 0, 0)
S10	(1, 0, 0, 0, 0)	(0.676, 0.324, 0, 0, 0)	(0.870, 0.130, 0, 0, 0)	(0.754, 0.246, 0, 0, 0)	(0, 0.304, 0.696, 0, 0)
S11	(1, 0, 0, 0, 0)	(0.501, 0.499, 0, 0, 0)	(0.900, 0.100, 0, 0, 0)	(0.782, 0.218, 0, 0, 0)	(0, 0.539, 0.461, 0, 0)
S12	(1, 0, 0, 0, 0)	(0.300, 0.653, 0.047, 0, 0)	(0.890, 0.110, 0, 0, 0)	(0.693, 0.307, 0, 0, 0)	(0.040, 0.349, 0.611, 0, 0)
S13	(1, 0, 0, 0, 0)	(1, 0, 0, 0, 0)	(0.882, 0.118, 0, 0, 0)	(0.758, 0.242, 0, 0, 0)	(0, 0.527, 0.473, 0, 0)
S14	(1, 0, 0, 0, 0)	(0.315, 0.685, 0, 0, 0)	(0.916, 0.084, 0, 0, 0)	(0.769, 0.231, 0, 0, 0)	(0, 0.581, 0.419, 0, 0)
S15	(1, 0, 0, 0, 0)	(0.936, 0.064, 0, 0, 0)	(0.921, 0.079, 0, 0, 0)	(0.776, 0.224, 0, 0, 0)	(0, 0.526, 0.474, 0, 0)
S16	(1, 0, 0, 0, 0)	(0.511, 0.489, 0, 0, 0)	(0.890, 0.110, 0, 0, 0)	(0.770, 0.230, 0, 0, 0)	(0, 0.528, 0.472, 0, 0)
